# Surface Conditioning Effects on Submerged Optical Sensors: A Comparative Study of Fused Silica, Titanium Dioxide, Aluminum Oxide, and Parylene C

**DOI:** 10.3390/s23239546

**Published:** 2023-11-30

**Authors:** Zibin Nan, Pascal Floquet, Didier Combes, Claire Tendero, Mickaël Castelain

**Affiliations:** 1 TBI, Université de Toulouse, CNRS UMR5504, INRAe UMR792—INSA 135, avenue de Rangueil, 31055 Toulouse, France; 2 LGC, Université de Toulouse, CNRS, INPT, UPS—ENSIACET 4, allée Émile Monso, 31030 Toulouse, France; pascal.floquet@toulouse-inp.fr; 3 CIRIMAT, Université de Toulouse, CNRS, INPT, UPS—ENSIACET 4, allée Émile Monso, 31030 Toulouse, France; claire.tendero@inp-toulouse.fr

**Keywords:** serum protein adsorption, polystyrene microbeads adhesion, shear stress flow chamber, principal component analysis, X-ray photoelectron spectroscopy

## Abstract

Optical sensors excel in performance but face efficacy challenges when submerged due to potential surface colonization, leading to signal deviation. This necessitates robust solutions for sustained accuracy. Protein and microorganism adsorption on solid surfaces is crucial in antibiofilm studies, contributing to conditioning film and biofilm formation. Most studies focus on surface characteristics (hydrophilicity, roughness, charge, and composition) individually for their adhesion impact. In this work, we tested four materials: silica, titanium dioxide, aluminum oxide, and parylene C. Bovine Serum Albumin (BSA) served as the biofouling conditioning model, assessed with X-ray photoelectron spectroscopy (XPS). Its effect on microorganism adhesion (modeled with functionalized microbeads) was quantified using a shear stress flow chamber. Surface features and adhesion properties were correlated via Principal Component Analysis (PCA). Protein adsorption is influenced by nanoscale roughness, hydrophilicity, and likely correlated with superficial electron distribution and bond nature. Conditioning films alter the surface interaction with microbeads, affecting hydrophilicity and local charge distribution. Silica shows a significant increase in microbead adhesion, while parylene C exhibits a moderate increase, and titanium dioxide shows reduced adhesion. Alumina demonstrates notable stability, with the conditioning film minimally impacting adhesion, which remains low.

## 1. Introduction

Optical sensors, renowned for their exceptional performance, face potential efficacy challenges when submerged due to the susceptibility of the outer surface to biological colonization [1,2,3]. This colonization, in turn, can lead to signal deviation, necessitating the development of robust solutions to ensure sustained accuracy and functionality. The protection of environmental sensors [2] is an important issue in so far as such sensors have to operate continuously and autonomously in immersion during several months (at least around 3 months) to collect high quality data. While immersed they undergo aggressions such as biofouling; i.e.,: microbial adhesion and biofilm formation, and therefore they have to be protected. Biofouling, denoting the colonization of microorganisms or macroorganisms on solid surfaces within aqueous environments [4], transitions into a biofilm during the stage of microorganism adhesion. A biofilm constitutes a structured community of bacterial populations enclosed in a self-produced polymeric matrix, firmly adhering to an inert or living surface [5,6]. The prevalence of biofouling and biofilm formation raises significant concerns across various domains, notably in marine sectors [7], food industries [8], medical contexts [9,10], and environmental monitoring [11,12]. Consequently, a substantial body of research has been dedicated to exploring antifouling strategies in recent decades, with a pronounced focus on environmentally sustainable alternatives following the prohibition of tributyltin compound applications in antifouling coatings in 2003 [13,14,15]. Most identified or strategies under research focus on prevention or denaturation of biofilm in the early stage of formation, mainly by avoiding the adhesion of particles or cells from the environment on the studied surface. These strategies include the application of nanomaterials, which benefits from their physical properties, such as silver nanoparticle [16,17], titanium dioxide (${\mathrm{TiO}}_{2}$) [18,19,20,21,22,23], and zinc oxide [24,25,26,27,28,29], the modification of surface with protein-resistant polymers [30,31,32], or slippery liquid-infused porous method [33] or self-assembled monolayers, which is able to control the adhesion through steric repulsion [34,35,36,37,38] and the immobilization of hydrolytic enzymes on the studied surface [39,40,41,42,43]. Despite the effectiveness of certain strategies, limited research has provided comprehensive elucidation of the molecular and physical interactions occurring during the adhesion process. As delineated in the existing literature, the mechanism of adhesion interaction is predominantly influenced by foundational determinants [44], including surface hydrophobicity/hydrophilicity [45,46,47,48,49], surface roughness [49,50,51], surface charge [45,52,53], chemical composition of the surface or adsorbate, such as functional groups [46,54], and the electrolytic environment [55,56,57]. In a general sense, the phenomenon of microbial adhesion is typically preceded by the establishment of a conditioning film [58,59,60] comprising macromolecular constituents. Through the adsorption of organic particles, conditioning films form on the substrate surface, potentially modifying the surface properties and thereby facilitating microbial adhesion [59,61,62,63]. The constituents of these conditioning films can vary significantly based on the environmental context to which the surface is exposed, thereby influencing the adhesion behavior of microorganisms, either augmenting or attenuating it [62,63,64].

In the context of environmental observatory and the imperative to devise novel strategies for mitigating biofouling on autonomous optical sensors deployed in continental waters, this study is dedicated to probing the nexus between physicochemical properties of coated or nanostructured surfaces and their propensity for adhesion of calibrated solid particles. Furthermore, the investigation extends to scenarios involving the presence of model conditioning proteins. The main goal is to propose some anti-fouling surfaces to prevent the adsorption of protein and provide a link to subsequent initial adhesion of particles.

To address this inquiry, four distinct specimens (namely fused silica, titanium dioxide, alumina, and parylene C) were subject to comprehensive characterization encompassing roughness, surface zeta potential, hydrophilicity, and surface chemical composition. For the sake of methodological simplicity, models were employed to emulate conditioning films, exemplified by Bovine Serum Albumin (BSA), as well as microorganisms represented by functionalized hydrophobic polystyrene microbeads. Subsequently, the detachment profiles of charged microbeads—denoting the count of adhered particles capable of withstanding shear flow relative to the applied shear stress—were assessed utilizing shear stress flow technique. These evaluations were conducted on both unaltered specimens and those subjected to BSA conditioning. Ultimately, the correlation among these findings was scrutinized through Principal Component Analysis (PCA).

## 2. Materials and Methods

### 2.1. Materials

To guarantee the best signal on immersed optical sensors, fused silica is a widely used material as the interface between the sensor and the environmental medium. Fused silica ($f-{\mathrm{SiO}}_{2}$) raw samples, NEGS1 quality, dimensioned for flow chamber (30 × 9 × 0.9 mm^3^) and for zeta potential (29.9 × 19.9 × 0.35 mm^3^) measurements, were purchased from Neyco and BSA purchased from Sigma Aldrich (Sigma-Aldrich Chimie, Saint-Quentin-Fallavier, France)). Functionalized polystyrene microbeads were purchased from Spherotech (Spherotech, Inc., Lake Forest, IL, USA). Two functions were chosen, amine groups ${\mathrm{NH}}_{2}$ (6.2 ± 0.8 μm in diameter) and carboxyl groups COOH (6.8 ± 0.7 μm in diameter). The diameters of the microbeads were measured using Malvern Mastersizer 3000 (Malvern Panalytical, Palaiseau, France) and fit to the specifications of the manufacturer. The suspending medium was Evian^TM^ (Danone, Rueil-Malmaison, France) water purchased from the supermarket. Evian^TM^ water has shown very similar physico-chemical properties as continental water in terms of ionic strength and pH, and the same batch was tested throughout the experiments presented in this paper. The electrical conductivity and pH was measured at 23 °C as σ = 534 ± 30 μS.cm${}^{-1}$ and pH = 7.2 ± 0.1, respectively. The total amount of dissolved solids was provided by the manufacturer at 345 mg/L. Ionic strength is approximated to *I* = 9.1 ± 0.5 mM. These values fit the drinkable water standards and to the majority of superficial natural waters in France (for instance, a real-time monitoring database, as in Ref. [65]).

### 2.2. Preparation of Samples

Titanium dioxide (${\mathrm{TiO}}_{2}$, anatase as crystalline structure), Aluminum oxide (${\mathrm{Al}}_{2}O_{3}$, amorphous), and Parylene C ${\left(C_{8}H_{7}\mathrm{Cl}\right)}_{n}$ coatings were deposited on fused silica by chemical vapor deposition. The depositions are presented in Table 1. Further details can be found in the referenced works. Before deposition process and further characterizations, fused silica samples were cleaned with ethanol, then rinsed in deionized water and dried under argon flow at room temperature.

### 2.3. Conditioning Film

BSA was chosen as the model protein to form the conditioning film on surface. BSA solution was prepared with a concentration of 1 g/L in Evian^TM^ water to model the ionic strength of real immersion media. In Evian^TM^ water, the ionic strength is 6.3 mM. Samples were immersed in BSA solution for 4 h to favor protein adsorption, and were then left dehydrating at room temperature under laminar flow hood, atmospheric pressure, and room temperature.

### 2.4. Polystyrene Microbeads

In order to understand the influence of coatings, polystyrene microbeads functionalized with carboxyl group (−COOH) or amino group ($-{\mathrm{NH}}_{2}$) have been chosen as the model of microorganism due to their similar size to microorganisms such as yeast cells or microalgae (5 to 7 μm in diameter) [69], their controllable density and non-pathogenic character. Moreover, carboxyl and amino groups are fundamental functional groups in protein and carry negative and positive charge, respectively: therefore, they can also be useful for assessing the protein adsorption mechanism. A volume of 100 μL original stock polystyrene microbeads was dispersed in 900 μL Evian^TM^ water and vortexed, then centrifuged at 13,000 rpm for 6 min. The precipitate was dissolved in 1 mL Evian^TM^ water and sonicated before use.

### 2.5. Atomic Force Microscopy (AFM)

The surface topography and roughness of bare surface samples were analyzed using an atomic force microscope (AFM, Agilent 5500^TM^, SCIENTEC, Les Ulis, France), in taping mode with regular Si (N-type) tips that have the following physical characteristics: curvature lower than 10 nm, stiffness between 25 and 75 N/m and resonance frequency in the 200–400 kHz range. Quadratic roughness calculation result from the analyses of 2 × 10 images (scanned area: 1 μm${}^{2}$) recorded on two specimens of each surface.

### 2.6. Water Contact Angle

The water contact angle of samples was investigated using Evian^TM^ water as sessile droplet via goniometer (DigiDrop^TM^, GBX, Romans-sur-Isère, France) on both bare and BSA-conditioned samples. For each surface, 10 measurements were performed, and each surface was triplicated. The conditioned samples were preliminarily immersed in BSA solution during 4 h.

### 2.7. Surface Charge

The surface charge of carboxyl group and amino group functionalized polystyrene microbeads and BSA molecule in the concentration of 1 g/L, were measured in 1 mM ${\mathrm{KNO}}_{3}$ solution using Zetasizer nano series (Malvern Instruments, Saclay, France). Zeta potential ζ of the flat samples surface (19.9 × 29.9 × 0.35 mm^3^) was measured with ZetaCAD^®^ setup (CAD Instruments, Les Essarts Le Roi, France). A volume of 1-mM ${\mathrm{KNO}}_{3}$ electrolyte solution was forced to pass through a capillary that consists of 2 similar flat samples separated by 200 μm-thick polytetrafluoroethylene spacers. The excess charges around the surface are carried along by the electrolyte. Their accumulation downstream results in an electric field that drives an electric current back by ionic conduction, through the liquid, against the direction of the liquid flow. A steady state is quickly established and the potential difference (between upstream and downstream) is measured with silver electrodes: this potential difference is called the streaming potential ΔE [70,71,72]. This streaming potential is linked to the zeta potential ζ (Equation (1)) through the Helmholtz–Smoluchowski approximation [73] that takes into account the physicochemical characteristics of the measurement medium:

(1) $$\frac{\Delta E}{\Delta P}=\frac{\epsilon_{0}\epsilon_{r}\zeta }{\mu \chi },$$

where ΔP is the charge loss in the streaming channel (Pa), ΔE the streaming potential (V), $\epsilon_{0}$ the vacuum permittivity (F.m${}^{-1}$), $\epsilon_{r}$ the solvent relative dielectric constant relative, ζ the zeta potential (V), μ the dynamic viscosity of electrolyte solution (Pa.s), and χ the conductivity of electrolyte solution (S.m${}^{-1}$). Before measurement, the samples were immersed in the electrolyte for 1 h for stabilization. In the case of BSA conditioning, samples were first immersed for 4 h in the BSA solution. The zeta potential was measured on both bare and BSA-conditioned surfaces. Each measurement was triplicated.

### 2.8. X-ray Photoelectron Spectroscopy (XPS)

Surface chemical composition was analyzed on both bare and conditioned substrates by performing X-ray photoelectron spectroscopy (XPS) measurements with a spectrometer (Thermo Scientific^TM^ Kalpha^TM^, Thermo Electron SAS, Toulouse, France) equipped with a monochromatic anticathode Al (Kα radiation of 1486.7 eV), under less than 5 × 10${}^{-9}$ mbar pressure. The energy calibration was performed using Au4f_7/2_ (83.9 ± 0.1 eV) and Cu2p_3/2_ (932.7 ± 0.1 eV) photoelectron lines. Charging compensation and neutralization was applied using a dual beam flood gun. The diameter of probed area was ∼400 μm. Each surface was characterized with two spots and duplicated. High-resolution spectra fitting was performed using NIST (https://srdata.nist.gov/xps/, accessed on 15 January 2023) database [74]. Conditioned samples were immersed in BSA solution, then rinsed with EvianTM water and dried with Argon whereas the bare substrates were only immersed in Evian^TM^ and dried with Argon. The comparison allows the investigation on BSA adsorption separated from the contribution of the immersion in water. As XPS analyses the extreme surface (the first 10 nm), the immersion time in BSA solution or Evian^TM^ water was set to 10 min in order to characterize the earliest steps of BSA adsorption and highlight the potential differences between the various surface behaviors and adsorption kinetics. Additionally, a 24-h immersion was tested in order to enhance the trends revealed during the first 10 min and favor the conditioning film formation in the case of very low adsorption kinetics.

### 2.9. Shear-Stress Flow Chamber

Shear flow-induced detachment experiments were performed to measure the adhesion of microorganisms on surfaces [55,75] or model particles [45,69] using a dedicated setup [45]. Polystyrene microbeads were selected as models of microorganisms. The flow chamber consisted of a rectangular channel of a very small cross section in which the flow could be kept laminar. The wall shear stress, $\tau_{w}$ (Pa), was thus perfectly controlled and it was assumed to be uniform over the whole coupon, i.e., the substrate surface. The previously described experimental procedure [75] was slightly modified, notably for the cell-counting mode. In brief, shear-flow induced microbeads detachment was analyzed in a rectangular flow channel (9 mm width, 30 mm length, and 200 µm thickness). The wall shear stress $\tau_{w}$ can be expressed as follows:

(2) $$\tau_{w}=\frac{3\mu Q}{4h^{2}l},$$

where *Q* is the volumetric flow rate (m${}^{3}$/s), μ is the dynamic viscosity (Pa.s), *h* is the channel half height (m), and *l* is the channel half width (m). An upright optical microscope (Nikon Eclipse LV100, Nikon Europe B.V., Amstelveen The Netherlands) equipped with a 20× working distance objective was set to observe experiments under the reflection mode, and a camera (digital STGHT DS-2MBW, Nikon Europe B.V., Amstelveen The Netherlands) with the NIS-Elements F3.0 video acquisition software was applied to record the image. Before starting the experiment, the flow chamber and all tubes were fulfilled with Evian^TM^, while all air bubbles were removed thoroughly from the system. The microbead suspension (with a volume of 1 ml at about $10^{6}$ particles/mL) was slowly injected into the flow chamber and then the beads could settle and attach to the substrate surface for 30 min under static condition. The initial number of adhered microbeads was recorded as $N_{0}$. A laminar flow was then imposed, with a stepwise increased flow rate ranging the corresponding wall shear stress $\tau_{w}$ from 0 to 80 Pa. At the end of each step, the number of microbeads remaining adherent to the surface (*N*) was counted. The detachment profile ($N/N_{0}=f\left(\tau_{w}\right)$) was then plotted (not shown). Experiments were performed in duplicate both on bare and BSA-conditioned samples (after 4 h immersion).

### 2.10. Principal Component Analysis

Principal component analysis (PCA) is a statistical tool that is used to reduce the dimensionality of large datasets, by transforming the set of variables into a smaller one that still contains most of the information in the large set ([76]). It reveals the correlations between variables and provides efficient plots that facilitate the interpretation. Datasets (individuals or variables) are represented in a new coordinate system consisting of ‘principal components’ (PCs), i.e., the axes of variability of the initial scatter graph. The raw data is converted into a standardized set of data by using the mean value and standard deviation. These standardized data are then compared with each other, leading to a correlation matrix. The matrix diagonalization results in a new set of coordinates named as *principal components*. Those principal components lead to different types of information:

- **Score plot:** The samples are plotted as a function of the new coordinate system and it can help to group samples with similar characteristics. ${\mathrm{Cos}}^{2}\theta$ calculated from the coordinates of the samples gives information on the quality of the sample description in the reduced space and thus the reliability of the drawn conclusion: the closer $\cos^{2}\theta$ is to one, the better the description;
- **Loading plot**: The variables are plotted as a function of the principal coordinates. Usually, two or three PCs are sufficient to take into account a large part of the data variability. Vectors that represent each variable should be drawn from the center: if there is, approximately, a ∼90° difference between the vectors, then the two variables are not correlated, while a difference of $∼180^{{}^{\circ}}$ means that the two variables are anti-correlated and if the angle is small (approximately ∼0–30°) between the two vectors then they are strongly related.

## 3. Results

### 3.1. Roughness and Topography

**Figure 1:**
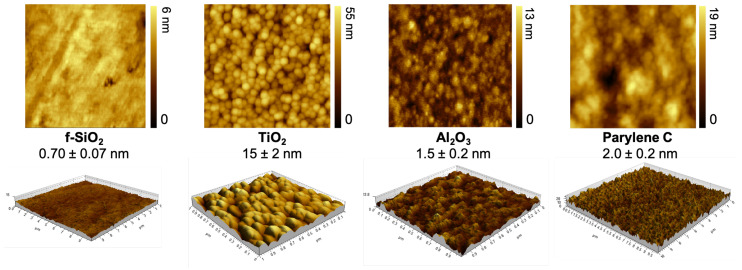
AFM mapping of surface topography within a 1-μm${}^{2}$ area and measurement of quadratic roughness (nm).

AFM technique was used to map out the surface topography and evaluate the quadratic roughness of the four investigated materials as presented on Figure 1. Fused silica is the flattest one with a 0.7 nm average roughness. Titanium dioxide exhibited the roughest topography on a nanoscale level, about 20 times higher than fused silica. The quadratic roughness of aluminum oxide and parylene C was between fused silica and titanium dioxide, respectively, being twice and three times higher than fused silica.

### 3.2. Hydrophobicity

**Figure 2:**
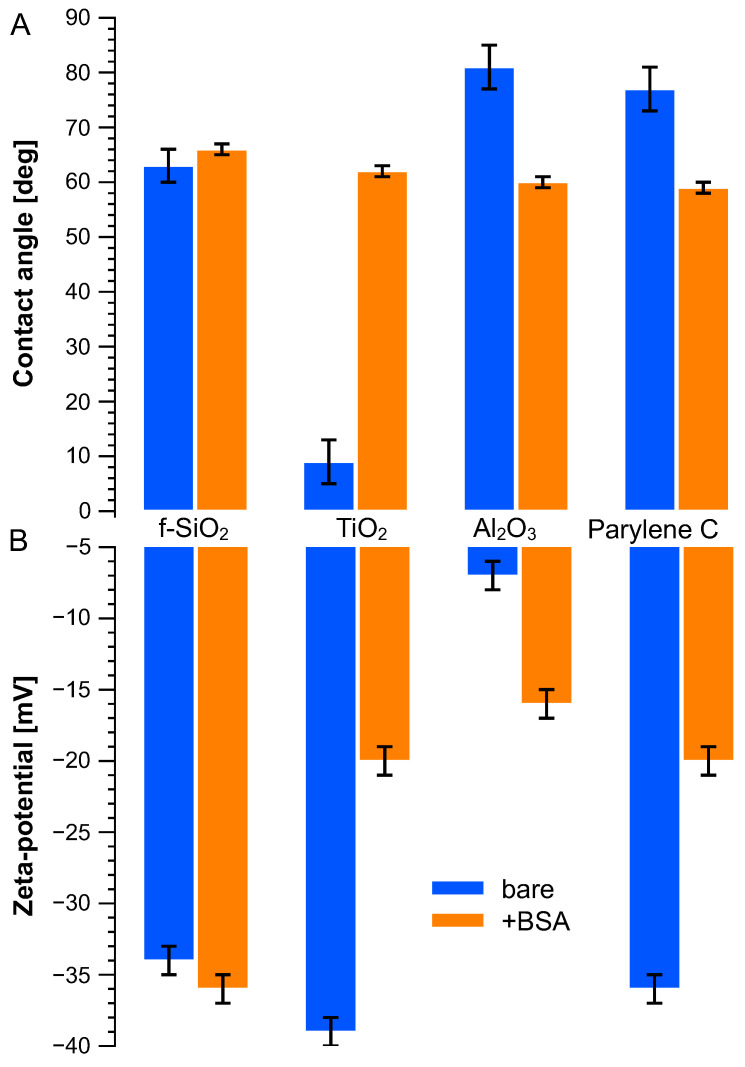
(**A**) Water contact angle and (**B**) surface zeta potential measured on bare surface (blue bars) and BSA-treated (orange bars) surfaces.

Figure 2A displays the water contact angle on these various surfaces, bare and after immersion in the BSA solution. Titanium dioxide showed a high hydrophilicity, whereas aluminum oxide and parylene C demonstrated similar hydrophobicity with water contact angles around 80°. When treated with BSA solution, water contact angles from these surfaces were around 60 degrees. The BSA molecule drastically increased the hydrophobicity of titanium dioxide, from (9 ± 4) to (62 ± 3) degrees and decreased slightly compared to that of aluminum oxide and parylene C. BSA immersion had limited influence on fused silica.

### 3.3. Zeta Potential

The zeta potentials of charged microbeads were measured as −110 mV and +14 mV, respectively, for carboxyl microbeads and amine microbeads under pH = 7. As for the BSA molecule, its zeta potential was measured as −25 mV. The zeta potentials of the flat surface were measured using streaming potential method and are plotted in Figure 2B according to Equation (1). All surfaces show negative potential whether they were immersed in BSA or not. Titanium dioxide has the highest surface zeta potential (−39 ± 1 mV) but remains close to fused silica (−34 ± 1 mV) and parylene C (−36 ± 1 mV), while aluminum oxide has the smallest zeta potential (−7 ± 1 mV). Once treated with BSA solution, their surface charges evolved to a similar level (ca. −20 ± 1 mV) except for fused silica, which remained stable at −36 ± 1 mV.

### 3.4. Chemical Composition by XPS

**Figure 3:**
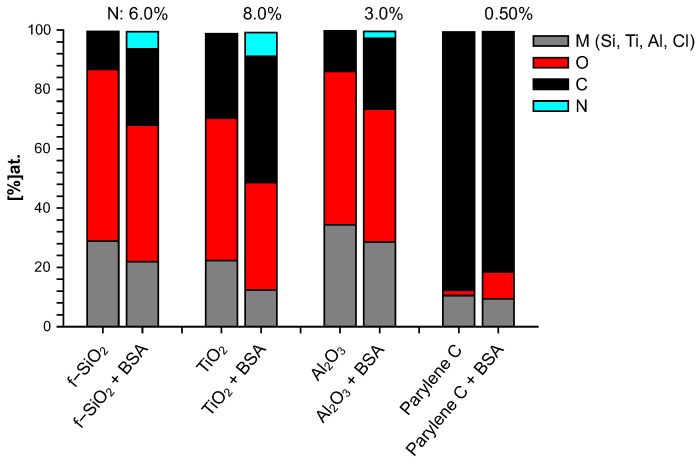
Chemical composition (% at.) of surfaces immersed 10 mn in water (bare) and 10 mn in BSA solution (+BSA).

The elemental composition of both bare and BSA-conditioned surfaces was determined by XPS: atomic ratios (%) are presented in Figure 3. It has to be mentioned again that the bare surfaces were analyzed after 10 mn immersion in water only. These bare surfaces exhibit a stoichiometric ratio of their constitutive elements with a few percent of residual carbon/oxygen contamination: ${\mathrm{TiO}}_{2}$, being a particularly reactive surface, presents the highest level of superficial contamination. No significant nitrogen was detected on these bare surfaces. Since BSA molecule is the only source of nitrogen, the additional proportion of nitrogen after immersion in BSA solution is a good indicator to track the quantity of BSA molecule adsorbed on the surface and therefore to investigate the protein adhesion ability of these surfaces. From this criterion, it can be concluded that after 10 mn immersion in BSA solution, the most important BSA adsorption occurs on titanium dioxide, followed by fused silica, aluminum oxide, and finally very poorly on parylene C. Nevertheless, the parylene C surface was modified by a significant enrichment in oxygen that was not observed after 10 min in water alone and could thus come from adsorption of the altered BSA. By “altered BSA”, we mean adsorption of BSA that is oxygen-richer than expected. This may be due to further adsorption of hydroxyl groups that is favored by the presence of BSA since this trend is not observed when bare parylene C is immersed in water.

**Figure 4:**
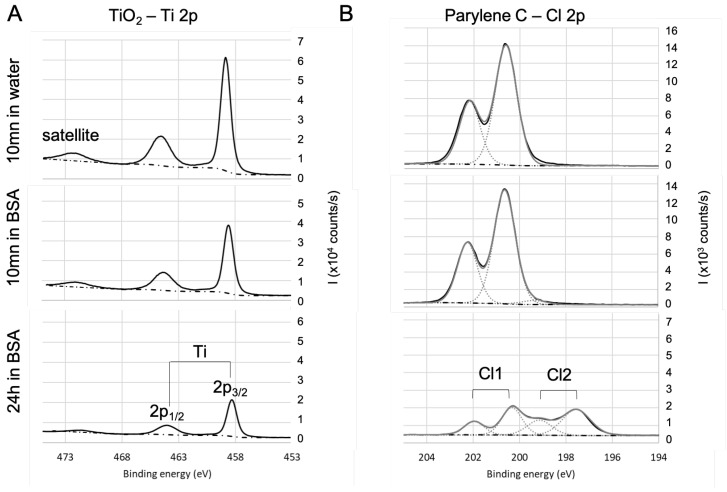
High-resolution XPS spectra of the heteroatom for (**A**) ${\mathrm{TiO}}_{2}$ and parylene C (**B**). Evolution with the immersion in BSA solution.

**Figure 5:**
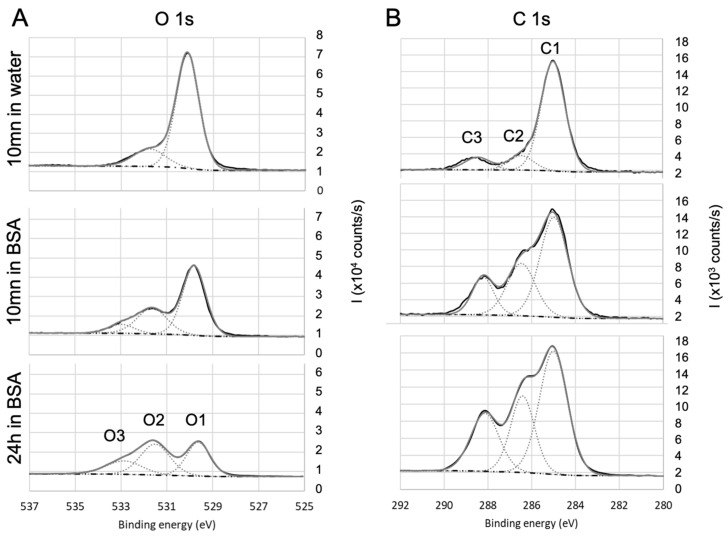
High-resolution XPS spectra of (**A**) oxygen and (**B**) carbon for ${\mathrm{TiO}}_{2}$. Evolution with the immersion in BSA solution.

**Figure 6:**
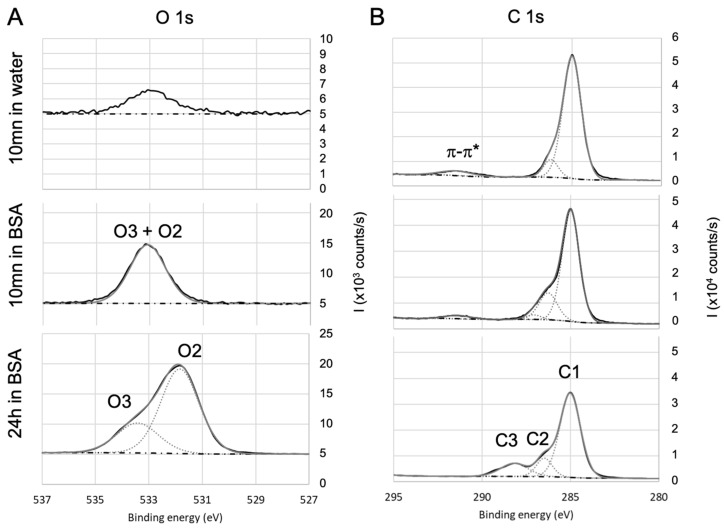
High-resolution XPS spectra of (**A**) oxygen and (**B**) carbon for parylene C. Evolution with the immersion in BSA solution.

**Figure 7:**
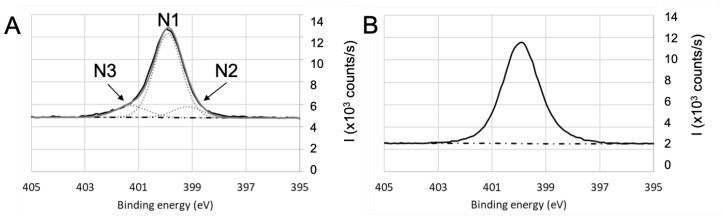
High-resolution XPS spectra of nitrogen for (**A**) ${\mathrm{TiO}}_{2}$ and (**B**) parylene C after a 24 h-immersion in BSA solution.

To further investigate the adsorption of BSA and draw some hypothesis on the mechanisms, the high-resolution spectra of the various single elements were then analyzed. The objective is to identify (i) the contribution of BSA in the various spectra and (ii) the nature of the bonds that are likely to be created between BSA and the different surfaces. To facilitate the deconvolution of the spectra, an additional immersion duration in BSA solution (i.e., 24 h) was tested: this led to the increase in the protein adsorption (as shown by the evolution of nitrogen contents presented in Table 2) and in the exaltation of the trends that were initiated during the first 10 min of immersion. Results for oxides (${\mathrm{SiO}}_{2}$, ${\mathrm{TiO}}_{2}$, ${\mathrm{Al}}_{2}O_{3}$) and non oxides (parylene C) are detailed as follow: a first focus on hetero atoms Ti to illustrate the oxides and Cl for Parylene—Figure 4, then on carbon and oxygen in the case of oxides (Figure 5) and parylene (Figure 6), to finish with nitrogen on both oxides (${\mathrm{TiO}}_{2}$) and parylene (Figure 7).

The spectra of Ti2p core level (see Figure 4A) for ${\mathrm{TiO}}_{2}$ samples are in agreement with the literature [77]: they exhibit a 5.7 ev spin-orbit split, a satellite structure around 472.3 eV, and $2p_{3/2}$ is located at 458.3 eV. The area ratio $2p_{3/2}:2p_{1/2}$ is 2:1. There is no significant evolution of this signature with the immersion in BSA solution except for the decrease in the intensity of the titanium signal. This decrease is due to the BSA adsorption on ${\mathrm{TiO}}_{2}$ that lowers the contribution of the ${\mathrm{TiO}}_{2}$ in the detected signal. As the Ti−O bond already exists on the bare surface, it is difficult to conclude on the existence of a bond between titanium and oxygen from BSA. The results (not shown here) are similar in the case of the Si2p and Al2p for respectively fused silica and alumina except for the fact that $2p_{3/2}$ and $2p_{1/2}$ are in the same envelope because the spin-orbit split is smaller. In the case of parylene C (see Figure 4B), Cl $2p_{3/2}$ of the bare sample is located at 200 eV, which is consistent with chlorine in the organic compounds, with a 1.6 eV spin-orbit split and a 2:1 area ratio of $2p_{3/2}:2p_{1/2}$. After 24 h immersion in BSA, Cl 2p exhibits another contribution (Cl2, with the same split and surface ratio as Cl1) at lower binding energy (197.6 eV for $2p_{3/2}$), which can be attributed to BSA adsorption with a bond that is likely to be formed between chlorine and BSA amine group.

The analysis of oxygen signal was then correlated with the one of carbon. In the case of ${\mathrm{TiO}}_{2}$ surface (Figure 5A), the oxygen spectra exhibited three contributions of O1 (529.6 eV), O2 (531.5eV), and O3 (532.5 eV), which can respectively be attributed to Ti−O, O=C, and O−C. The O1 atomic ratio is consistent with the stoichiometry of titania and, as the titanium signal, decreases with the immersion in BSA solution.

O2 and O3 contributions are confirmed by the analysis of carbon C1s core-level high-resolution spectra (Figure 5B). The main component of C1sis located at 285 eV and corresponds to aliphatic carbon: C−C and C−H bonds (C1). After immersion in BSA, the contributions at 286.6 eV (C2) and 288.6 eV (C3) respectively attributed to C−N, C−O, and O=C−O, O=C−N are significantly increased, confirming the presence of BSA (peptide bonds) adsorbed on the surface of titanium oxide. Moreover, the ratio %N/(%C1+%C2) is close to 0.5 once the residual contamination observed on bare surface is deduced, which is in agreement with previous work [78,79]. For fused silica and alumina, a similar conclusion can be drawn.

For parylene C, the O1s core level high-resolution spectra (Figure 6A) exhibit only O2 and O3 contributions after 24 h immersion in BSA, confirming the presence of adsorbed BSA. O2 and O3 also exist after 10 min immersion in BSA, but are less marked. On the carbon spectra (Figure 6B), C1 also includes C−C aromatic and C−Cl. The component at 291.5 eV is attributed to the satellite structure induced by $\pi -\pi^{*}$ excitations on the aromatic ring [80]. After 10 mn immersion in BSA, C2 is increased compared to the bare surface and can be correlated to the increase in oxygen O2 + O3. Therefore, it can be concluded that after 10 min immersion in BSA, the surface of parylene C is enriched in oxygen: this may be due to adsorption of altered BSA due to adsorption of both BSA and hydroxyl groups as (i) the amount of adsorbed nitrogen does not match with stoichiometric BSA, (ii) C3 is too small to confirm peptide bonds and (iii) the O2+O3 contribution was lower after 10 min immersion in water alone. After 24 h immersion, the amount C2 and C3 are in agreement with O2 and O3.

N1s high-resolution spectra (Figure 7) after immersion in BSA exhibit N1 contribution (located at 400 eV), which is consistent with amine group (C−N bonds). N2 contribution attributed to C=N can be located in the 398–399 eV range. In the case of parylene C (Figure 7B), N3 contribution was attributed to the $N_{\mathrm{BSA}}-{\mathrm{Cl}}_{\mathrm{Parylene}}$ bond, which is in agreement with the Cl2 contribution observed on Figure 4B and confirmed previously by assigning this N3 contribution at 402.5 eV to $-{\mathrm{NH}}_{3}^{+}$ [69]. For titanium oxide, shown on Figure 7A, it is difficult to sharply conclude on a contribution of a $O_{\mathrm{sample}}-N_{\mathrm{BSA}}$ bond because of the shape and symmetry of the N1s signal. If present, it would be around the high binding energy tail of the signal. The same observation can be made for fused silica and aluminum oxide.

**Figure 8:**
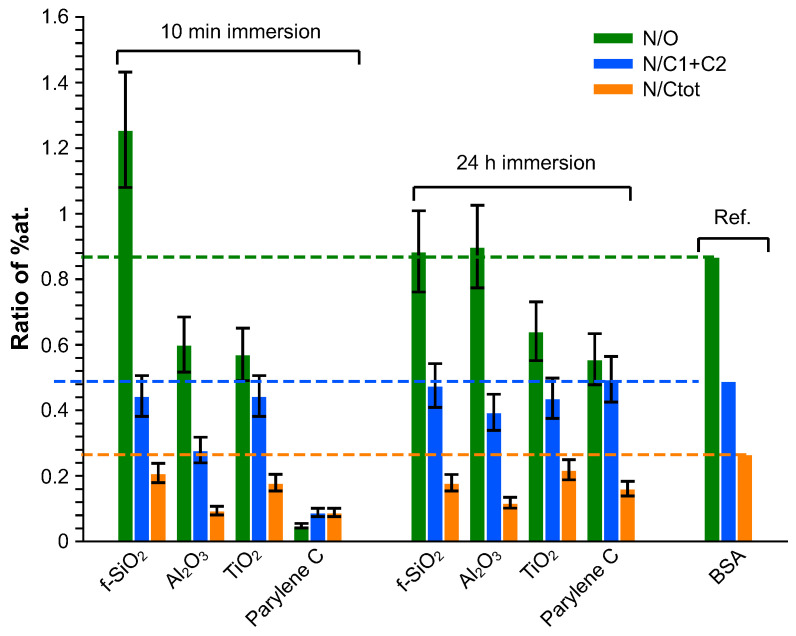
Elemental composition of the adsorbed layer.

The composition of the adsorbed layer was finally investigated in terms of N/O, N/(C1 + C2) and N/C ratios and compared to BSA stoichiometric composition [81]. The contribution of the bare substrate was subtracted from the global quantitative analysis, considering that the composition of the substrate was stoichiometric: the evaluation of this contribution was deduced from the amount of the heteroatom. The results are presented in Figure 8. Globally, whatever the immersion duration, all the adsorbed layers exhibit an excess in the carbon amount. After 10 min immersion, the N/O ratio is higher than the reference on silica fuse and lower on alumina and titanium dioxide. Considering that the N/(C1+C2) ratio matches with the reference for $f-{\mathrm{SiO}}_{2}$ and ${\mathrm{TiO}}_{2}$, this means that the adsorbed layer is oxygen-deficient for $f-{\mathrm{SiO}}_{2}$ and presents an excess in oxygen on ${\mathrm{TiO}}_{2}$. On alumina, the superficial layer is nitrogen-deficient. After 24 h immersion, except for the excess on contamination carbon, the adsorbed superficial layer is rather close to BSA composition on fused silica and alumina whereas it remains too rich in oxygen on parylene C and titanium dioxide.

### 3.5. Microbeads Adhesion to Surfaces

Shear-induced detachment of the two types of beads was monitored by video microscopy. The wall shear stress required to remove half the initial population $\tau_{w}^{50}$ is reported in Table 3 according to the surfaces tested and the type of microbead. Therefore, the lower $\tau_{w}^{50}$ value, the better anti-adhesion performance. When this value is not reached, meaning that more than 50% of microbeads were still on the surface in the end of experiment, the surface exhibits adhesive properties. When no detachment occurs, this value can reach 80 Pa (the maximum value obtained in this setup).

For both microbeads, with positive or negative charge, titanium dioxide showed significant adhesion to surfaces, higher than the three other materials, and fused silica exhibited the smallest $\tau_{w}^{50}$ value. Besides, negatively charged microbeads adhere steadier on the surface than positively charged microbeads, in general. When treated with 1 g/L BSA solution, the performance of these materials varies: the adhesion of both microbeads remarkably increased on fused silica and decreased on aluminum oxide. However, the situation tends to be different on titanium dioxide and parylene C. On titanium dioxide, adsorbed BSA reduced the adhesion of positively charged microbeads and did not affect that of negatively charged microbeads. On parylene C, adsorbed BSA slightly reduced the adhesion of positively charged microbeads but enhanced that of negatively charged microbeads.

## 4. Discussion

This study delves into the investigation of how Bovine Serum Albumin (BSA), employed as a model protein for conditioning immersed surfaces, adsorbs under low ionic strength [79]. The research is conducted within the framework of immersed monitoring optical sensors, with X-ray Photoelectron Spectroscopy (XPS) serving as the primary analytical tool for scrutinizing the adsorption of BSA onto various materials [79], aiming to mitigate biofouling concerns. To fortify our findings, we employed microbeads functionalized with carboxyl or amine groups within a shear-flow chamber [45,69,75]. This approach allows for a comprehensive evaluation of adhesion properties. Furthermore, Principal Component Analysis (PCA) was implemented and presented in this section to elucidate potential correlations between substrate and microbead surface properties.

### 4.1. Analysis of BSA Adsorption

The adsorption of BSA was investigated by plotting the data presented in Table 4. Not-reached values of $\tau_{p}^{50}$ were estimated and ranked from the detachment profiles in order to allow the PCA calculation.

**Figure 9:**
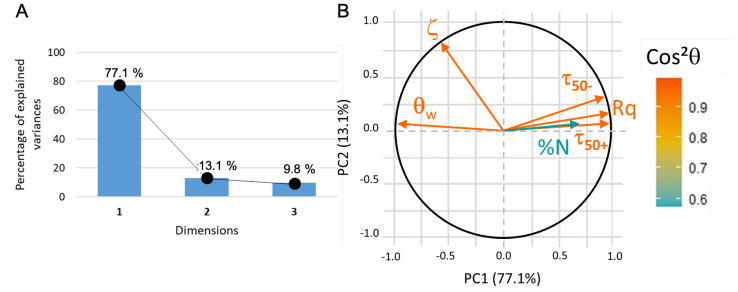
(**A**) PCA1 variance plot that shows that 90% of the information is described by PC1 and PC2. (**B**) Loading plot where $cos^{2}\theta$ indicates that only protein adsorption data is not correctly represented.

For this first PCA analysis, all the variables are active (as they participate to the PC building). The data are correctly represented (about 90% of the information, see Figure 9A) in a 2D coordinate system where the first principal component (PC1) can be assimilated to roughness, water contact angle, and adhesion of functionalized beads whereas PC2 is correlated to zeta potential (Figure 9B). The nature of PC1 indicates that both roughness and hydrophilicity (i.e., small water contact angle) strongly favor the adhesion of microbeads (whether positively or negatively charged), whereas the ζ potential of substrates appears uncorrelated.

In the context of this 2D system, the adsorption of protein is partially elucidated and appears to closely align with the first principal component (PC1). Subsequently, an additional principal component analysis was conducted, omitting consideration of the zeta potential values due to their lack of correlation with the remaining dataset. This exclusion finds validation in the experimental observation that BSA adsorption takes place in immersion media characterized by a slightly higher ionic strength compared to that employed in zeta potential measurements for both microbeads and surfaces. It can be hypothesized that, during the BSA adsorption phase, the surface zeta potential of the immersed substrates is likely obscured by the presence of other charges in the solution and may not exert a significant influence. This observation is further supported by the empirical finding that negatively charged microbeads exhibited stronger adhesion to the substrates compared to positively charged counterparts, notwithstanding the negative zeta potential exhibited by all substrates.

**Figure 10:**
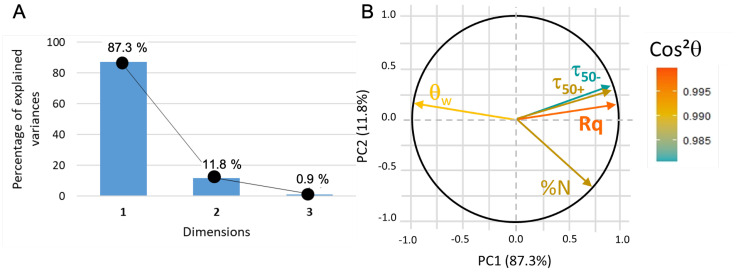
(**A**) PCA2 variance plot that shows that 98% of the information is described by PC1 and PC2. (**B**) Loading plot where $cos^{2}\theta$ indicates that all the data are correctly represented.

**Figure 11:**
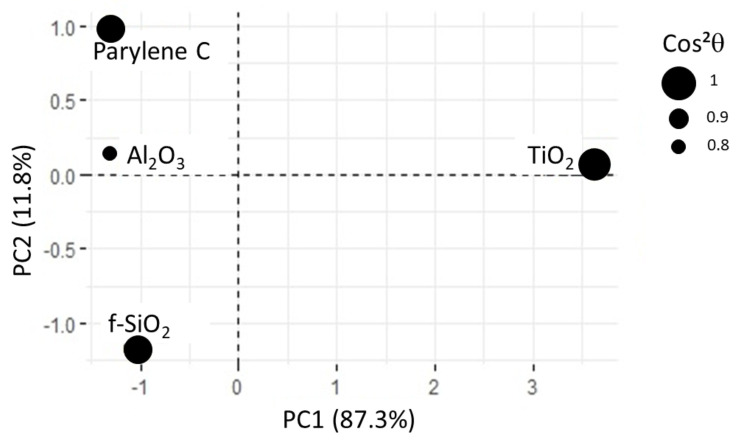
Score plot for PCA2. All the substrates are correctly represented, as shown by $\cos^{2}\theta$ values. For alumina surface, $\cos^{2}\theta$ is close to 0.8.

In this new plotting system, the adsorption of BSA is much better represented (Figure 10). It is positively linked to roughness and hydrophilicity, but also depends on another factor, PC2, which remains to be identified. The score plot positions ${\mathrm{TiO}}_{2}$ and ${\mathrm{Al}}_{2}O_{3}$ surfaces along PC1 while parylene C, ${\mathrm{Al}}_{2}O_{3}$ and $f-{\mathrm{SiO}}_{2}$ are along PC2 (see Figure 11). In other words, considering protein adsorption, alumina and titanium dioxide mainly differ in roughness and hydrophilicity whereas parylene C, ${\mathrm{Al}}_{2}O_{3}$ and $f-{\mathrm{SiO}}_{2}$ are essentially discriminated by PC2.

This first analysis on protein adsorption is in agreement with results from literature [82,83]. Surface roughness, in a nanometric range, as well as hydrophilicity, strongly influence protein adsorption, while zeta potential does not seem to be significant. An additional component is missing to further describe the interaction between surface and protein. The component that discriminates parylene C, alumina, titanium oxide, and fused silica could be correlated to the nature of the bonds involved in each material. The bonds drive the charge distribution at the surface and thus the reactivity and the adsorption sites. By considering the Pauling electronegativity of each element, fused silica is ∼60% covalent whereas aluminum oxide and titanium are rather ∼60% ionic: the electrons delocalization in ionic compounds favors reactivity and adsorption. Parylene C is mostly covalent, but the presence of polar chlorine atom induces a dipole: chlorine appears to be a preferential adsorption site, which was confirmed by XPS analysis. The lower adsorption rate on parylene C in the earlier adsorption steps is due to reduced number of adsorption sites in comparison with iono-convalent compounds. The importance of the surface charge distribution on protein adhesion was pointed out by Beragoui et al. [84]. They studied BSA adsorption on differently functionalized polystyrene surfaces, demonstrating the important contribution of hydrogen bonds in the adsorption, compared to hydrophobic and electrostatic interactions. Ab initio calculation coupled to adsorption isotherm experiments would be useful to further investigate the local charge distribution on each surface and the resulting adsorption scenario and kinetics.

### 4.2. Influence of the Conditioning Film on the Adhesion of Functionalized Hydrophobic Polystyrene Microbeads

To analyze the influence of BSA adsorption on the adhesion of the functionalized microbeads, PCA-3 was run in two steps, considering the data presented in Table 5. Once the appropriate coordinate system was found to describe the surfaces before immersion in BSA, the surfaces after immersion were plotted in this 2D system. In statistical terms, PCA-3 was run considering surface description before immersion in BSA as “active individuals” and surface description after immersion as “supplementary individuals”.

**Figure 12:**
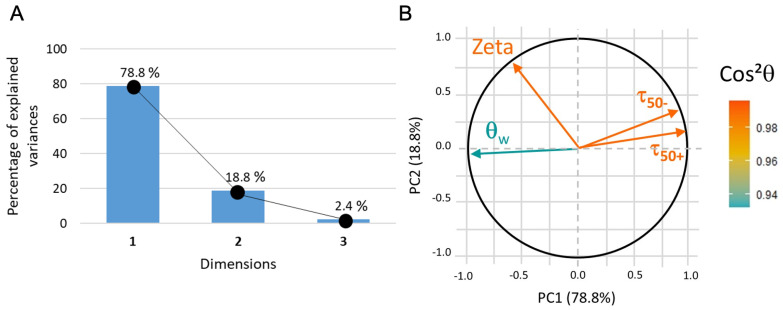
(**A**) PCA3 variance plot that shows that 97% of the information is described by PC1 and PC2. (**B**) Loading plot where $cos^{2}\theta$ indicates that all the data correctly represented.

The data plot in the 2D coordinate system (with hydrophilicity as PC1 and PC2 partially correlated to surface zeta potential, see Figure 12) indicates that, without conditioning film, the adhesion of microbeads, whether positively or negatively charged, is mainly driven by hydrophilicity. Nevertheless, the adhesion is systematically stronger in the case of carboxyl-microbeads.

**Figure 13:**
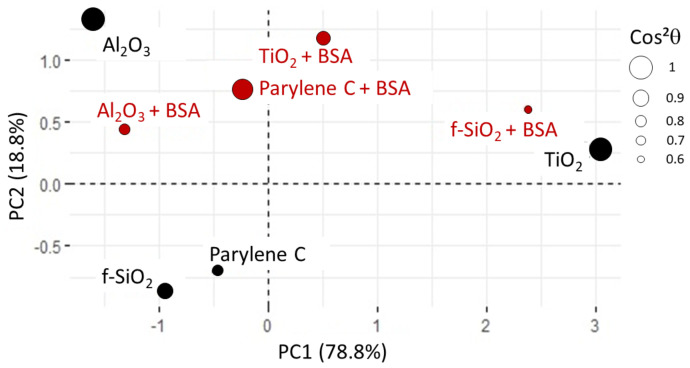
Score plot for PCA3. The $Cos^{2}\theta$ values shows that the representation of fused silica in BSA is the least reliable (0.6 for $f-{\mathrm{SiO}}_{2}$). All the other samples are correctly represented with $cos^{2}\theta$ ranging from 0.76 to 1.

After immersion of the surfaces in BSA, the evolution of the adhesion of microbeads depends on the surface, as illustrated by the score plot (Figure 13). All the sample are correctly represented by PC1 and PC2, except for $f-{\mathrm{SiO}}_{2}$ in BSA, which presents 0.6 as $cos^{2}\theta$ and a significant coordinate on PC3. Anyway, the comparison of $f-{\mathrm{SiO}}_{2}$ before and after immersion in BSA remains reliable as the change in the coordinate along PC1 and PC2 is quite significant. On ${\mathrm{Al}}_{2}O_{3}$ and parylene C, the adhesion is poorly affected, whereas it is drastically increased on $f-{\mathrm{SiO}}_{2}$ and significantly decreased on ${\mathrm{TiO}}_{2}$. This behavior is observed for both functional groups, but it is emphasized in the case of carboxyl-microbeads. This can be explained by the iono-covalent nature of bonds: on covalent fused-silica, the protein conditioning film creates polar adsorption sites (−COO− and $-{\mathrm{NH}}_{3}^{+}$). On aluminum oxide, titanium dioxide and parylene C, BSA is likely to create polar sites on a surface that already had some. Therefore, no change on microbeads adhesion is observed on aluminum oxide and parylene C. In the case of titanium dioxide, adhesion property is reduced because of the modification of hydrophilicity, which is in line with protein adsorption. The immersion of the surfaces in BSA reduces the dispersion of the samples regarding PC2, which could be the correlation between zeta potential and the iono-covalent nature of bonds. Finally, as the BSA-samples do not have all the same coordinates, we can assume that the immersed surface are different: in other words, they are either not fully covered by BSA or BSA adhered in different conformations, as suggested in previous works [85]. Devilopoulos et al. [86] also observed that BSA adsorbed differently regarding the nature of the substrate: dense layers are formed on parylene C whereas thicker and more diffuse layers develop on silicon oxide. Further analysis with FTIR and Raman microscopy as well as AFM adhesion force mapping need to be performed to characterize the coverage homogeneity as well as the BSA conformation [87].

### 4.3. Towards the Adsorption of Microorganisms

As alluded to above, the primary objective of this investigation is to elucidate the adsorption behavior of Bovine Serum Albumin (BSA), employed as a model protein, in order to assess its interaction with modified surfaces, with the ultimate aim of proffering potential strategies for anti-fouling surface development. The empirical foundation of this inquiry rests upon an in-depth examination of microbead adhesion properties under controlled flow conditions, subsequently correlated with pertinent surface properties. In light of these achievements, the subsequent phase of our research endeavors will entail subjecting these surfaces to microbial organisms—an aspect not within the immediate purview of this study, yet one that merits discussion in the ensuing section and represents a prospective avenue for further inquiry.

The conclusion on the influence of hydrophilicity on microbeads adhesion is consistent with results on hydrophobic bacteria from literature. Some earlier studies pointed out that substrate surface hydrophobicity is an important factor for bacterial adhesion, where a superhydrophobic surface significantly reduced the adhesion of some hydrophobic bacteria such as *Escherichia coli*, *Staphylococcus aureus*, and *Pseudomonas aeruginosa* [88,89,90]. By using hydrophobic bacteria *E. coli*, Friedlander et al. [91] observed an increase of bacterial adhesion as polydimethylsiloxane (PDMS) surface status changing from non-wetting to wetting. Lu et al. [92] also demonstrated a decrease of *E.coli* coverage rate due to the surface becoming more hydrophobic. On the contrary, Thewes et al. [93] did a single-cell force spectroscopy study with hydrophilic bacteria *Staphylococcus carnosus*, indicating that unspecific bacterial adhesion is governed by hydrophobic interaction and decreases as the surface became more hydrophilic. This hydrophilic repulsion, which was subjected by hydration pressure [73] between microorganisms and surfaces, displaying hydrophilic properties, was also pointed out previously, with yeast cells experiencing a decrease of such repulsion with an increase in ionic strength [94].

In addition to hydrophobicity concerns, in aqueous environments, the prevailing forces are typically Lifshitz–van der Waals (LW) and electrostatic (EL) forces, as outlined in [95] following the DLVO theory, which stands for Derjaguin–Landau–Verwey–Overbeek. Given that microorganims and most synthetic and natural surfaces carry a negative charge at typical pH levels, electrostatic interactions are generally repulsive. At low ionic strength (1 mM), the long-range DLVO-type electrostatic repulsion takes precedence over van der Waals attraction [94]. However, under high ionic strength conditions, van der Waals attraction becomes dominant [57,95,96,97]. Nonetheless, the DLVO theory has only seen limited success in elucidating microbial adhesion phenomena on diverse surfaces. This is due to its failure to account for non-DLVO interactions, such as hydrophobicity and hydrophilicity, as expounded by the extended DLVO theory [73,98,99,100,101,102].

However, consideration must be given that the composition of the microbial cell surface deviates markedly from that of a hydrophobic colloid. This surface is predominantly constituted of biopolymers, exhibiting variable degrees of solvation. Consequently, the intermolecular interactions occurring at this interface assume pivotal importance. These interactions may encompass the establishment of covalent bonds between specific segments of the macromolecular species and the substrate surface. Furthermore, microbial cells exhibit an active capacity to release macromolecules into the surrounding milieu. This dynamic process expeditiously conditions the substrate surface through adsorption as modeled in this project by BSA, thereby establishing a conducive environment for cellular adhesion [103]. Empirical validation of this phenomenon has been provided, specifically in the context of *Azospirillum brasilense* [104]. The interplay between the conditioned surface and microbial cells is further nuanced, potentially involving bridging interactions between segments of the macromolecular entities. Such interactions are notably potentiated by increased ionic strength and the presence of divalent cations. Moreover, specific interactions have been observed in instances of mammalian cell-substrate interactions, particularly when the substrate has been conditioned by protein adsorption [105].

Concerning the influence of surface nano-roughness on the adhesion of microorganisms, the literature remains conflicting. Some studies suggested that cell adhesion was favored on a nano-rough surface [106] whereas others showed a better adhesion in the case of a nano-smooth surface [107,108]. The impact of surface roughness has to be considered globally: $R_{a}$ or $R_{q}$ are not sufficient to correctly describe the topography: shapes, texturing, and aspect ratio should also be taken into account [109,110]. Vadillo-Rodriguez et al. [111] reported that the adhesion of both *Staphylococcus epidermidis* and *Staphylococcus aureus* was significantly reduced by engineered surface patterns with nanometer vertical dimensions (i.e., smaller than cell dimension), suggesting that the singular points designed on the surface (i.e., square corners, convex walls) drove the initial cell location and could further interfere with cell–cell communication and thus biofilm growth.

Finally, the impact of the conditioning film on cell adhesion is reported to be strongly dependent on the nature of the substrate that drives the formation of this conditioning layer, as well as the ionic strength of the immersion media. In Ref. [86], the authors showed that parylene C and silicon oxide, after immersion in the same protein serum, exhibit drastically different conditioning film, resulting in much higher cell adhesion on silicon oxide. This trend was also observed by Hwang et al. [62] for *Burkholderia cepacia* on BSA-coated silica slides but only at low ionic strength (in 1 mM KCl). At higher ionic strength, BSA conditioning film significantly hindered bacterial adhesion.

## 5. Conclusions

In the realm of environmental observatories and the pressing need to develop innovative approaches for reducing biofouling on autonomous optical sensors deployed in continental waters, this study focuses on exploring the relationship between the physico-chemical characteristics of coated or nanostructured surfaces and their susceptibility to the adhesion of precisely calibrated solid particles. Additionally, the inquiry encompasses situations where model conditioning proteins are present. The primary objective was to suggest anti-fouling surfaces that deter protein adsorption (BSA) and establish a connection to the subsequent initial adhesion of particles. Two simplistic functions were selected (carboxyl and amine), which are usually encountered on microorganisms.

In this context, four distinct surfaces (fused silica, titanium dioxide, aluminum oxide, and parylene C) were evaluated both before and after being immersed in BSA solution. The assessment involved parameters such as roughness, surface charge, water contact angle, surface composition of the substrates, and interaction with functionalized microbeads, which served as models for simplified microorganisms.

The findings indicate that protein adsorption was primarily influenced by surface roughness at a submicrometric scale, hydrophilicity, and likely connected to the electron distribution on the surface, hence, the type of bonds present on the material surface. Among the surfaces, titanium dioxide exhibited the highest adsorption properties, while parylene C showed a lower kinetic rate.

In terms of protein adsorption mechanisms, clear evidence of bonds between amine groups and chlorine is observed on parylene C. However, on the oxide, there is no conclusive indication of the formation of Moxide-OBSA or Ooxide-NBSA bonds, or both. Both scenarios are plausible, and further investigation through ab initio and molecular dynamic calculations is needed.

After a 24-h immersion in BSA solution, alumina and silica developed a conditioning film with a composition closely resembling that of BSA, whereas an excess of oxygen was noted on titanium dioxide and parylene C.

This conditioning film had varying effects on surface interactions with microbeads, influencing hydrophilicity as well as the local distribution of charges. Fused silica experienced a substantial increase in microbead adhesion, while parylene C showed a milder effect, and titanium dioxide displayed reduced adhesion. Alumina distinguished itself for its stability, as the growth of the conditioning film has a limited impact on its adhesion properties, which remain relatively low.

To comprehensively assess the influence of the conditioning film on microbial adhesion, further studies need to be conducted, incorporating experiments involving relevant microorganisms.

## Figures and Tables

**Table 1:** Process parameters for the deposition of coatings, where *d* is the coating thickness, *P* and *T* are the operating pressure and temperature for deposition, respectively.

Coating	Precursor	Deposition Parameters	Reference
${\mathrm{TiO}}_{2}$	Titanium isopropoxide	*P* = 5 mbar	[66]
*d*∼300–400 nm	Bubbling at 50 °C under nitrogen flow	*T* = 400 °C	
${\mathrm{Al}}_{2}O_{3}$	Aluminium isopropoxide	*P* = 5 mbar	[67]
*d*∼300 nm	Direct liquid injection	*T* = 500 °C	
Parylene C	dichloro[2,2]paracyclophane	*P* = $10^{-2}$ mbar	[68]
*d*∼300 nm	Sublimation at 140 °C then pyrolysis at 670 °C	*T* room temperature	

**Table 2:** Evolution of the nitrogen content (at%) of the four substrates with the immersion duration in BSA solution.

Immersion Time	0	10 min	24 h
$f-{\mathrm{Sio}}_{2}$	0.20 ± 0.02	6.0 ± 0.2	10 ± 1
${\mathrm{TiO}}_{2}$	0.45 ± 0.05	8.0 ± 0.2	11 ± 1
${\mathrm{Al}}_{2}O_{3}$	0.10 ± 0.02	3 ± 1	7 ± 1
Parylene C	0.20 ± 0.05	0.50 ± 0.05	9 ± 1

**Table 3:** $\tau_{w}^{50}$ value of bare and BSA-treated (+BSA) surfaces measured in shear stress flow chamber using positively charged (${\mathrm{NH}}_{2}$(+)) and negatively charged (COOH(-)) polystyrene microbeads. In case the detached number of beads did not reach down the half population, $\tau_{w}^{50}$ displays n/a.

$\tau_{w}^{50}$ (Pa)	NH${}_{2}$(+)		COOH(-)	
	**bare**	**+BSA**	**bare**	**+BSA**
f-${\mathrm{SiO}}_{2}$	1.1 ± 0.3	23 ± 7	5 ± 3	n/a
${\mathrm{TiO}}_{2}$	17 ± 4	9 ± 4	n/a	n/a
${\mathrm{Al}}_{2}O_{3}$	2 ± 1	1.1 ± 0.3	24 ± 9	9 ± 3
Parylene C	5 ± 2	3 ± 2	22 ± 6	n/a

**Table 4:** Dataset used for the principal components PCA1 and PCA2. $\tau_{w}^{50-}$ and $\tau_{w}^{50+}$ represent the $\tau_{w}^{5}0$ of carboxyl and manie microbeads, respectively.

Surface	$R_{q}$ (nm)	$\theta_{w}\left({}^{\circ}\right)$	ζ (mV)	$\tau_{w}^{50+}$ (Pa)	$\tau_{w}^{50-}$ (Pa)	%N (%at.)
$f-{\mathrm{Sio}}_{2}$	0.7 ± 0.1	63 ± 3	34 ± 1	1.1 ± 0.3	5 ± 3	6.0 ± 0.2
${\mathrm{TiO}}_{2}$	15 ± 2	9 ± 4	−39 ± 1	17 ± 4	80 ± 3	8.0 ± 0.2
${\mathrm{Al}}_{2}O_{3}$	1.5 ± 0.2	81 ± 4	−7 ± 1	2 ± 1	24 ± 9	3 ± 1
Parylene C	2.0 ± 0.2	77 ± 3	−36 ± 1	5 ± 2	22 ± 6	0.50 ± 0.05

**Table 5:** The dataset used for PCA3. The first step consists of determining the PCs, and the second step determines the influence of the BSA surface conditioning.

PCA3	Surface	$\theta_{w}\left({}^{\circ}\right)$	ζ (mV)	$\tau_{w}^{50+}$ (Pa)	$\tau_{w}^{50-}$ (Pa)
1st step	$f-{\mathrm{Sio}}_{2}$	63 ± 3	−34 ± 1	1.1 ± 0.3	5 ± 3
	${\mathrm{TiO}}_{2}$	9 ± 4	−39 ± 1	17 ± 4	80 ± 3
	${\mathrm{Al}}_{2}O_{3}$	81 ± 4	−7 ± 1	2 ± 1	24 ± 9
	Parylene C	77 ± 3	−36 ± 1	5 ± 2	22 ± 6
2nd step + BSA	$f-{\mathrm{Sio}}_{2}$	66 ± 4	−36 ± 1	23 ± 7	80 ± 3
	${\mathrm{TiO}}_{2}$	62 ± 4	−20 ± 1	9 ± 4	65 ± 3
	${\mathrm{Al}}_{2}O_{3}$	60 ± 4	−16 ± 1	1.1 ± 0.3	9 ± 3
	Parylene C	59 ± 6	−20 ± 1	3 ± 2	50 ± 3

## Data Availability

All data are available upon request.

## Abbreviations

The following abbreviations are used in this manuscript:

AFM	Atomic Force Microscopy
BSA	Bovine Serum Albumin
${\mathrm{TiO}}_{2}$	Titanium dioxide
$f-{\mathrm{SiO}}_{2}$	Silica fuse
${\mathrm{Al}}_{2}O_{3}$	Alumina
XPS	X-ray Photoelectron Spectroscopy
